# Exploring Restrictions to use of community greenways for physical activity through structural equation modeling

**DOI:** 10.3389/fpubh.2023.1169728

**Published:** 2023-07-18

**Authors:** Erkang Fu, Xiaoyu Deng, Yuanhao Wu, Lin Li, Yilin Xiong, Mengzhou Li, Zihan Zhang, Juan Du, Xinyun Li, Xi Li

**Affiliations:** College of Landscape Architecture, Sichuan Agricultural University, Chengdu, China

**Keywords:** community greenway, physical activity, restriction, structural equation modeling, landscape characteristics

## Abstract

Public health problems caused by rapid urbanization have attracted increasing amounts of attention. Existing studies show that improving the frequency and duration of physical activity among urban residents can effectively reduce their disease risk. A community greenway, as a green space for public activity directly serving community residents, is one of the best spatial place for bringing health benefits to people. Although the scale and scope of greenway construction have been increasing in recent years, the utilization rate of some greenways is not high for various reasons, restricting the extent to which people engage in healthy physical activities in greenway spaces. In this study, the greenway of Nancheng Community in Wenjiang District, Chengdu city, China was selected as the object of study, and structural equation modeling was conducted to explore the objective environmental factors and individual characteristics acting as barriers to use of the community greenway by the population for physical activity. The results show that user experience, the greenway landscape, and safety and accessibility are important factors that restrict people’s willingness engage in physical activity in the community greenway environment. The results of this study provide a direction for further consideration of ways to enhance people’s willingness to make use of greenways for physical activity, and further provide a theoretical basis for the healthy design and transformation of community greenway spaces.

## 1. Introduction

Rapid urbanization has brought an increasing number of public health problems to the attention of the public and has increased health risks among the population in several ways, especially in the areas of mental illness (1, 2), chronic diseases such as cardiovascular disease (3, 4), and general health (5–7). Empirical studies of environmental health and public health indicate that increasing the frequency and duration of physical activity can effectively reduce the risk of these diseases among urban residents. For example, increasing physical activity as a form of leisure can improve the health of the population (8), enhance physical and mental health (9), and also help to reduce stress, regulate emotions, and improve cognition (10–13).

Green open space is an important feature of a healthy outdoor living environment and an important type of space for the promotion of people’s participation in physical activity. Research has found that the number of parks in the vicinity of an area of residence is directly proportional to the intensity of physical activity engaged in by its population, and the provision of different types of environment within a park can support different types and levels of physical activity (14). Residents’ personal attributes also play a moderating role in the relationship between green space and recreational physical activity (15). Some scholars have also focused on the relationship of green public space with physical activity among different groups: for example, community parks and trail length are positive predictors of increased physical activity among older adults (16), while street greenways also result in increased physical activity among older adult patients and in the creation of a healthy aging environment (17).

As an indispensable linear form of green open space and a component of an urban green space system (18, 19), greenways provide residents with a suitable space for slow walking and can be used as sports venues (20, 21); they therefore have the health-related effects of relieving mental pressure, increasing physical activity, and promoting social interaction. By creating an ecologically friendly environment, greenways can bring people closer to nature to relieve mental stress (22, 23), provide a walking environment for the promotion of physical activities such as walking, cycling, and stretching (24, 25), and form a network of green channels to connect different communities, thereby stimulating public interaction (26). According to empirical research, the above benefits are more evident in the case of greenways connecting neighboring communities (27, 28), which can positively impact and restore the mental state and physiological capabilities of residents (29), especially those of older adults in the community (30, 31). Greenways connecting neighboring communities affect the amount of exercise taken by residents, mainly through the provision of a pedestrian environment enabling community residents to take control of their engagement in physical activity; this benefits the amount of exercise they take, which in turn improves the health of residents (32). Thus, the features of greenways in the built environment can positively affect the intensity of residents’ physical activity (33).

Although the scale and scope of greenway construction have been increasing in recent years, the utilization rate of some greenways in reality is not high, as a result of many subjective or objective restrictions limiting individuals’ participation in healthy physical activities on the greenway space; this reduces the quality of recreation services provided by the greenway. Among these restrictions, intrapersonal, interpersonal, and structural restrictions all affect people’s use of greenways for recreational activities (34), and generally, these three factors impose decreasing levels of constraint, in the order of mention (35). Previous studies have proved that preference, time, travel costs, and geographical distance are the main factors restricting recreational activities in urban green spaces (36), but few researchers have discussed the factors specific to community greenways regarding the willingness of people to engage in physical activity.

**Figure 1:**
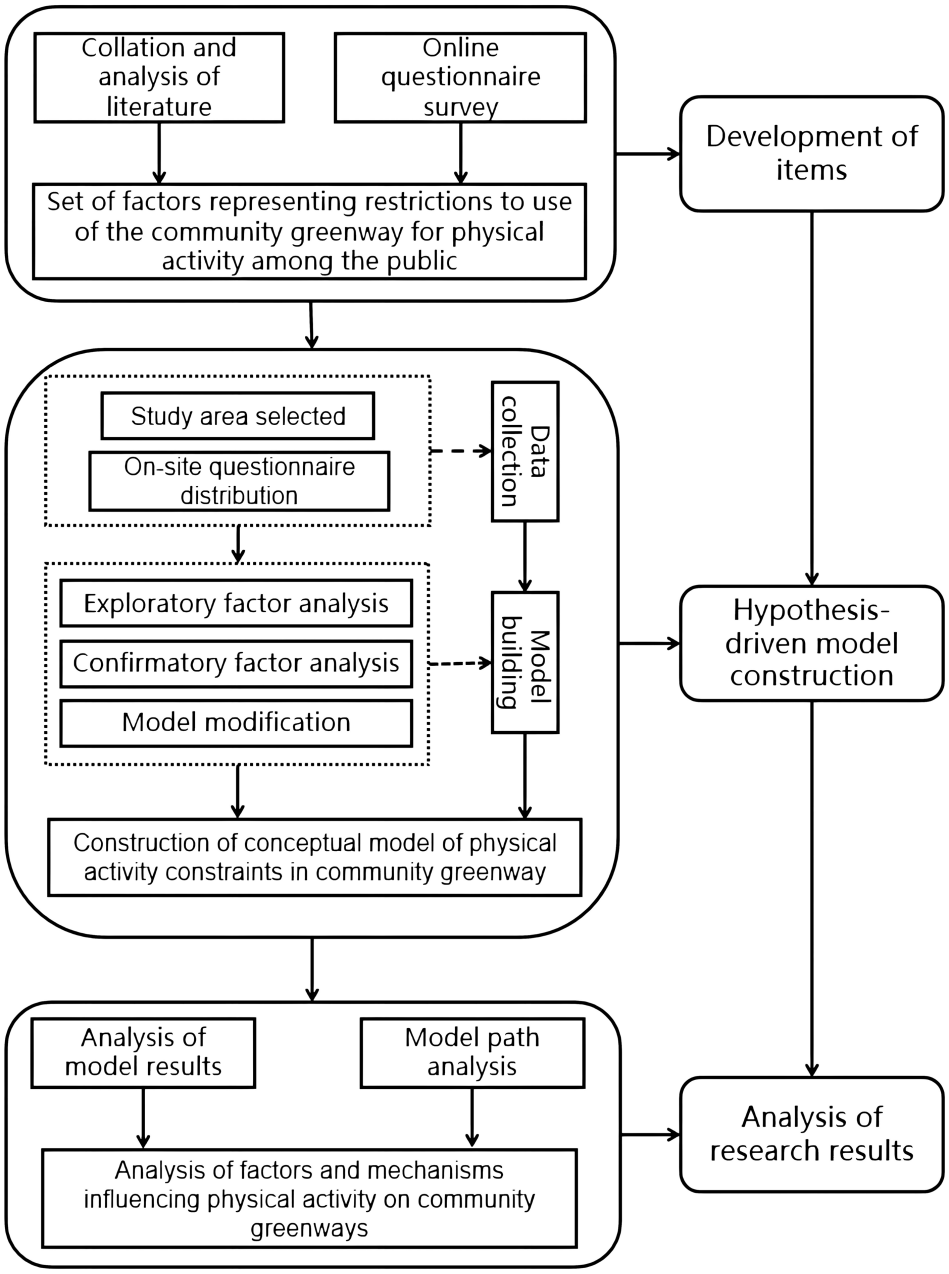
Flow chart for this study of restrictions to use of community greenways by the public for physical activities.

Therefore, on the basis of a literature review and questionnaire-based survey, the aim of this study was to construct a model of the factors acting as restrictions to the use of the community greenway for physical activity by the population. Taking the greenway of Nancheng Community in Wenjiang District of Chengdu as the object of this social investigation, structural equation modeling was used to verify the variables identified, with the aim of exploring the objective environmental factors and individual characteristics that act as restrictions to use of the community greenway for physical activity among the population, and of exploring the strength of each influencing factor (Figure 1).

## 2. Methods

### 2.1. Site selection

**Figure 2:**
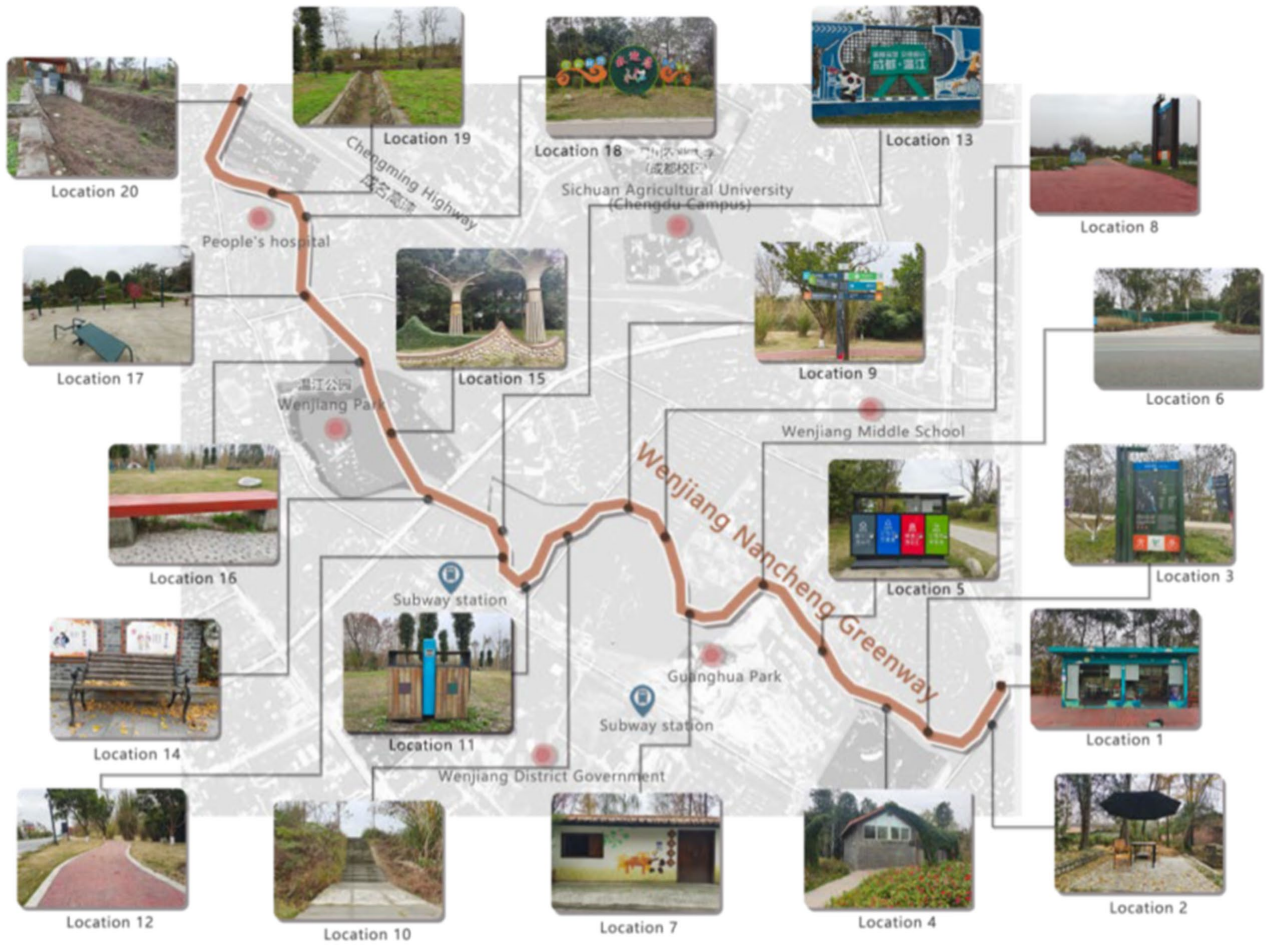
Schematic map of “green islands” in the southern district of Wenjiang.

The South City Community Greenway in Wenjiang District, Chengdu, China was selected as the research object for this study. This 87-km-long greenway connects schools, metro stations, bus stops, hospitals, and other public services constituting a 15-min living circle, providing a very good spatial place and a high-quality space for green and low-carbon travel, grocery shopping, leisure and sports, and neighborhood interaction for residents in the surrounding area; it is one of the most frequently used greenways in Chengdu. A total of 20 residential areas, clusters, and compounds within 1 km of the South City Community Greenway Station and its surrounding areas were selected for inclusion in this study (Figure 2).

### 2.2. Design of the questionnaire

#### 2.2.1. Initial selection of items

Following extensive reading and collation of literature related to greenways and constraints on them, 14 articles with strong relevance to the content of this study were identified. In-depth interpretation and analysis of these was carried out; relevant elements mentioned in the literature pertaining to the characteristics of greenways and personal subjective factors that restrict the willingness of the public to make use of them were extracted, and an initial set of factors constraining public use of community greenways was constructed (Table 1). This initial selection of factors included five factor dimensions as latent constructs: greenway landscape (GL), service facilities (SF), personal and interpersonal factors (PH), experience of use (UE), and accessibility and safety (SA). Each dimension contained several specific items, for a total of 32 observed variables. The five latent variables could not be measured through direct observation in practice, but the observed variables could be; therefore, the latent constructs in the factor system were measured via the corresponding observed variables.

**Table 1:** Research on restrictions to physical activity on greenways.

Author	Research methods	Influencing factors
Keith et al. (37)	Descriptive analysis; regression analysis	Population characteristics; motivations for using the greenway; website preferences
Senes et al. (38)	Linear regression analysis	Landscape features; time; accessibility; density of roads; topography; historical and cultural interest
Akter et al. (39)	Correlation analysis Multiple linear regression analysis	Distance from home; barrier-free structures; landscape; lighting; drinking water and toilet facilities; maintenance standards; cleanliness; pavement width; indicating system; availability of parking lot
Mundet and Coenders (40)	Statistical analysis	Seating/rest area; drinking water facilities; conflict with motor vehicles; hygiene; topography; lavatory facilities; tree cover for shade
Coutts (41)	Statistical analysis	Density of population; degree of land-use diversity
Lindsey (42)Yang et al. (43)	Regression analysis. The GIS network analysis method	Visual permeability; intercommunity links; greening rate; land-use diversity; proportion of paved roads; density of facilities; continuity, accessibility, environmental comfort, spatial diversity
Qiaoqiao and Fengquang (44)	Correlation analysis Linear regression analysis	Transportation; natural environment; environmental hygiene
Jiang et al. (45)	Importance–Performance Analysis (IPA)	Ecological and cultural landscape; accessibility; infrastructure; management and maintenance services; surface conditions
Ye (46)	Analytic hierarchy process (AHP)analysis	Road network planning; design of scenic spots; design of green corridors; indicating system; parking facilities; recreation facilities; environmental sanitation facilities
Liu (47)	Factor analysis	Supporting facilities; service facilities; stage; greening environment; accessibility
Lu et al. (48)	Correlation analysis Factor analysis	Accessibility; environmental landscape
Zhanqiang et al. (49)	Linear regression analysis	Density of population; degree of land-use diversity; neighboring settlements
Lu and Lu (50)	Statistical analysis	Environmental sanitation facilities; safety facilities; accessibility

#### 2.2.2. Optimization of items

Through interviews with people using the greenway and relevant experts, items were added to and removed from the set of potential influencing factors was added, items were categorized, and the latent variable of personal and interpersonal factors (PH) was added. On the basis of the initial set of items for evaluation, the “Questionnaire on Constraints on Physical Activity Among Community Greenway Users” was developed to optimize measurement of the relevant items, taking into account the purpose of this study. Responses were given on a Likert scale, with respondents indicating the strength of each of the barriers as one of five levels: no effect, weak effect, average effect, strong effect, or very strong effect. Each of the 34 items was evaluated separately. The optimized form of the questionnaire was finally established as shown in Table 2, consisting of 6 latent variables and 34 observed variables (Table 2).

**Table 2:** Preliminary selection of items and variables for the model of barriers to peoples’ use of the community greenway for physical activity.

Latent variables	Observed variables
Personal and interpersonal factors (PH)	My attitude toward fitness and sense of engagement (PH1)
	Attitudes of friends and family toward fitness (PH2)
	My psychological health (PH3)
	My physical health (PH4)
	Time occupied by work/family(PH5)
	Have worked together (PH 6)
Greenway landscape (GL)	Architectural style and shape of gardens (GL1)
	Logo esthetics (GL2)
	Planting of garden plants and shading effect (GL3)
Services and facilities (SF)	Point-of-sale setup (SF1)
	Provision of fitness and sports facilities (SF2)
	Installation and cleanliness of toilets and drinking fountains (SF3)
	Installation of streetlamps and other lighting (SF4)
Safety and accessibility (SA)	Presence of sharp bulges in the seats and other facilities (SA1)
	Absence of unsafe remote dead ends (SA2)
	Installation of guardrails in hazard zones (SA3)
	Motor vehicle parking (SA4)
	Blocking the condition of motor vehicle facilities (SA5)
	Surrounding traffic (SA6)
	Interference of pedestrians and bicycles with each other (SA7)
	Non-motor vehicle parking (SA8)
	Convenience of access (SA9)
User experience (UE)	Marker lines indicate the correct condition (UE1)
	Prominent positioning of marker lines(UE2)
	Convenience of sideway crossing (UE3)
	Signs and lines are simple and easy to understand (UE4)
	Legibility of jogging lanes (UE5)
	Barrier-free design (UE6)
	Connectivity of the greenway to attractions (UE7)
	Ease of crossing of overpasses and tunnels (UE8)
	Ease of transfer to public transport (UE9)
	Pavement design (UE10)
People’s willingness to engage in physical activity (AW)	Satisfaction with the greenway (AW1)
	Willingness to go to the greenway for physical activity (AW2)

### 2.3. Procedure

The survey was completed between October and December of 2021 by a random sample of participants. For some participants who encountered difficulties in reading and filling out the questionnaire, the survey was administered in interview form and the participants were assisted in filling out their responses according to their opinions. A total of 350 questionnaires were distributed and 322 valid sets of responses were returned, for a valid return rate of 92%. As shown in Table 3, the participants represented a wide range of ages, education levels, and occupations, with a high degree of randomness, ensuring the reliability of the findings of the analysis.

**Table 3:** Studies on physical activity restrictions in greenways.

Variable	Categories	Number	Percentage
Sex	Man	164	50.9%
	Woman	158	49.1%
Age	<6 years old	0	0
	7–12 years old	0	0
	13–17 years old	6	1.9%
	18–30 years old	272	84.5%
	31–45 years old	18	5.6%
	46–60 years old	12	3.7%
	61–75 years old	10	3.1%
	>75 years old	4	1.2%
Household structure	Living alone	46	14.3%
	Family of two	24	7.5%
	Family of three	48	14.9%
	Family of 4–5	28	8.7%
	Other	176	54.7%
Education level	Junior high school or below	14	4.3%
	High school/vocational school	12	3.7%
	Undergraduate/junior college	194	60.2%
	Postgraduate or above	102	31.7%
Occupation	Public institution/civil servant/government work	8	2.5%
	Professional (e.g., teacher/doctor)	16	5.0%
	Service staff (e.g., driver/shop assistant)	2	0.6%
	Worker (e.g., factory worker/sanitation worker)	0	0

### 2.4. Statistical analysis

Structural equation modeling (SEM) is a method for the construction, estimation, and testing of models of causal relationship; it is an extension of a variety of multivariate analysis techniques. A structural equation model contains both measurable observed variables and latent variables that cannot be directly observed. SEM can replace multiple regression, path analysis, factor analysis, covariance analysis, and other methods, and enables clear analysis of the effects of individual factors on the entire set of outcomes and the relationships between individual factors. Compared with traditional analysis methods, SEM enables explanation of as much of the variability as possible while providing an understanding of the covariant relationships between variables. There are two types of factor analysis within SEM: exploratory and confirmatory factor analysis. Exploratory factor analysis (EFA) is used to extract the structure of a set of data; CFA is used to validate hypotheses regarding observed and latent variables. In this study, EFA was first conducted to extract the main factors, and CFA was then used to validate the structure of the factors imposing constraints on physical activity on the greenway. On the basis of the structure arising from the CFA results, a model of barriers to physical activity on the community greenway is proposed.

## 3. Results

### 3.1. Construction of the conceptual model

#### 3.1.1. Analysis of validity

Before exploratory factor analysis (EFA) is conducted, the data should first be analyzed for reliability and validity. The reliability of the valid data obtained in the present study was analyzed using SPSS 22.0. Cronbach’s alpha coefficient for the standardized items was 0.953, indicating that the reliability of the questionnaire was high. The main tests of validity employed were Bartlett’s test of sphericity and the KMO test. The results are shown in Table 4: the KMO value was 0.885 (KMO > 0.60), indicating that there was no significant difference in the correlation degree of each variable. For Bartlett’s test of sphericity, *χ*^2^ = 8533.125, *p* = 0.000 (*p* < 0.001), indicating that the collected data for observed variables exhibited good intercorrelation and met the requirements for EFA analysis.

**Table 4:** KMO and Bartlett’s tests.

KMO test		0.885
Bartlett’s test of sphericity	Chi-square	8533.125
	DF	496
	Significance	0.000

#### 3.1.2. Exploratory factor analysis

Exploratory factor analysis (EFA) was first conducted to extract the dominant factors underlying restrictions to engagement in physical activity on the community greenway. Principal component analysis with varimax rotation was employed to determine the orthogonal factors, and factors with an eigenvalue greater than one were identified. As listed in Table 5, five factors were extracted based on EFA, accounting for approximately 66.26% of the total variance, with factor loadings ranging from 0.409 to 0.821. Common factor 1 represented “safety and accessibility,” explaining 21.94% of the variance; common factor 2 represented “user experience,” explaining 16.48% of the variance; common factor 3 represented “personal and interpersonal factors,” explaining 11.53% of the variance; common factor 4 represented “services and facilities,” explaining 9.09% of the variance; and common factor 5 represented “the greenway landscape,” explaining 6.61% of the variance.

**Table 5:** Summary of the results of EFA of restrictions to physical activity on the community greenway.

Underlying factors	Items	Cumulative variance explained	Factor loading coefficient
Safety and accessibility(SA)	Presence of sharp bulges in the seats and other facilities (SA1)	21.94%	0.821
	Absence of unsafe remote dead ends (SA2)		0.820
	Installation of guardrails in hazard zones (SA3)		0.811
	Motor vehicle parking (SA4)		0.804
	Blocking the condition of motor vehicle facilities (SA5)		0.801
	Surrounding traffic (SA6)		0.714
	Interference of pedestrians and bicycles with each other (SA7)		0.698
	Non-motor vehicle parking (SA8)		0.642
	Convenience of access (SA9)		0.578
User experience(UE)	Marker lines indicate the correct condition (UE1)	16.48%	0.802
	Prominent positioning of marker lines (UE2)		0.787
	Convenience of sideway crossing (UE3)		0.728
	Signs and lines are simple and easy to understand (UE4)		0.701
	Legibility of jogging lanes (UE5)		0.659
	Barrier-free design (UE6)		0.539
	Connectivity of the greenway to attractions (UE7)		0.509
	Ease of crossing of overpasses and tunnels(UE8)		0.506
	Ease of transfer to public transport (UE9)		0.435
	Pavement design(UE10)		0.409
Personal and interpersonal factors(PH)	My attitude toward fitness and sense of engagement (PH1)	11.53%	0.808
	Attitudes of friends and family toward fitness (PH2)		0.731
	My psychological health (PH3)		0.729
	My physical health (PH4)		0.723
	Time occupied by work/family (PH5)		0.650
	Have worked together (PH 6)		0.581
Services and facilities(SF)	Point-of-sale setup (SF1)	9.09%	0.748
	Provision of fitness and sports facilities (SF2)		0.690
	Installation and cleanliness of toilets and drinking fountains (SF3)		0.647
	Installation of streetlamps and other lighting (SF4)		0.574
Greenway landscape(GL)	Architectural style and shape of gardens (GL1)	6.61%	0.651
	Logo esthetics (GL2)		0.563
	Planting of garden plants and shading effects (GL3)		0.523

#### 3.1.3. Confirmatory factor analysis

The common factors extracted via EFA were taken as latent variables, and the items falling within these were taken as the corresponding observed variables; a measurement model in the form of a structural equation model was thus established. In order to further test the reliability of the measurement model, confirmatory factor analysis (CFA) was carried out on the measurement model, including reliability analysis and validity analysis. The reliability analysis was conducted by computing Cronbach’s α coefficient for each variable in the measurement model. As shown in Table 6, Cronbach’s α coefficient was greater than 0.8 for each of the six latent variables, and the overall Cronbach’s α coefficient was 0.953, indicating good reliability among the observed variables within each latent variable and among all latent variables, with good internal consistency.

**Table 6:** Reliability of latent variables.

Factor	Cronbach’s α	Number of items
Personal and interpersonal factors (PH)	0.847	6
Greenway landscape (GL)	0.814	3
Services and facilities (SF)	0.827	4
User experience (UE)	0.916	10
Safety and accessibility (SA)	0.944	9
People’s willingness to engage in physical activity (AW)	0.925	2
Overall	0.953	34

In SEM, in order to test whether the model achieves a good fit, it is generally necessary to conduct statistical analysis by calculating the ratio of the chi-square statistic to the respective degrees of freedom (*χ*^2^/DF), the RMSEA, the GFI, the CFI, and other indicators of fit. In this case, *χ*^2^/DF = 6.699, and the standard criterion value is 1–3, meaning that this measure indicated that the goodness of fit was not up to standard; RMSEA = 0.673, and this value should be <0.08, also indicating that the goodness of fit was not up to the standard; and GFI and CFI were calculated to be 0.633 and 0.673, respectively, while these two indicators should be >0.9 (Table 7). Therefore, the indicators of goodness of fitness did not reach the standard criteria, indicating that the model fit was inadequate, and the model needed to be adjusted and corrected.

**Table 7:** Analysis of initial model fit.

Goodness-of-fit measures	*χ*^2^/DF	GFI	CFI	RMSEA
Before model modification	6.699	0.633	0.673	0.673
Recommended range	1–3	>0.9	>0.9	<0.08

### 3.2. Construction of the structural equation model

**Figure 3:**
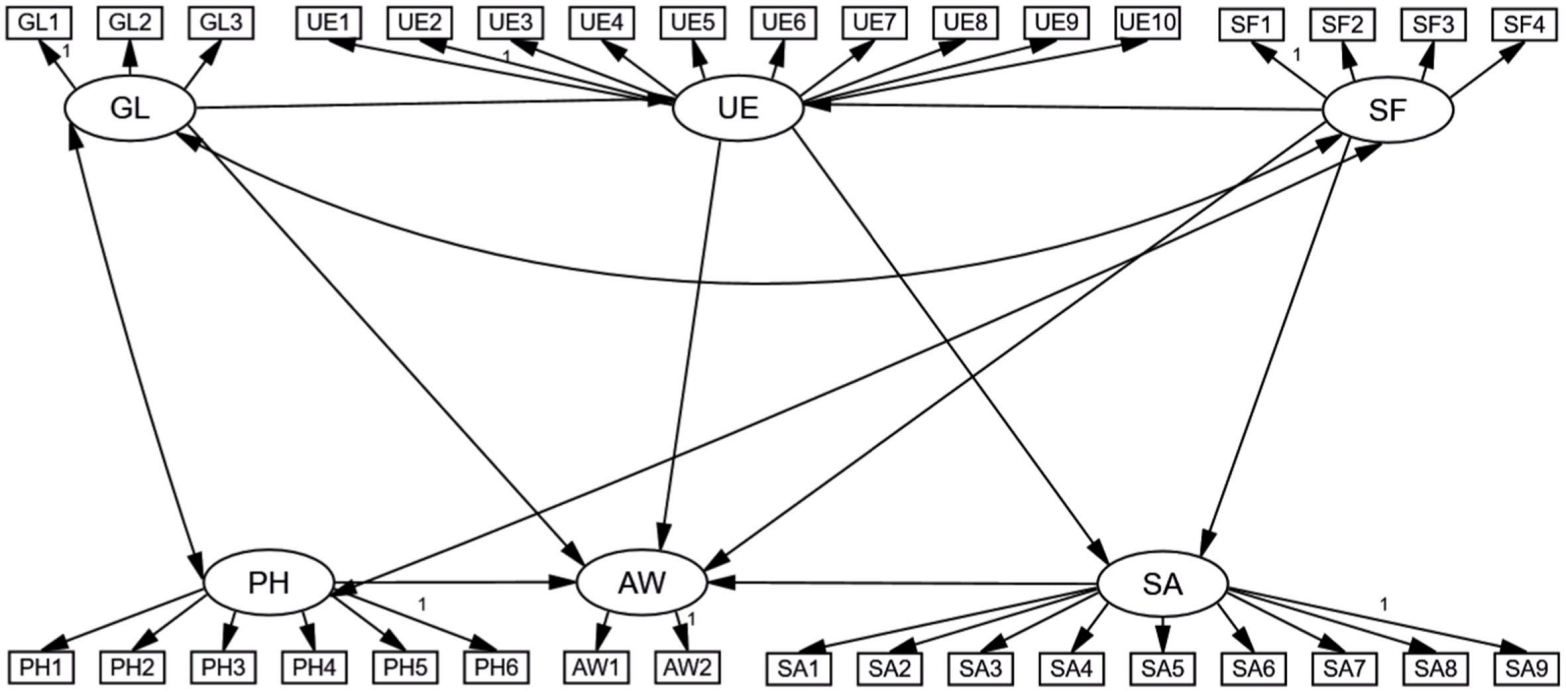
Initial model of factors restricting physical activity on the greenway among the population of the greenway community.

The conceptual structural equation model describing the relationships between the six latent variables is shown in Figure 3. The main specific hypotheses relating to barriers to physical activity in community greenways are presented as follows:

*H1*: Provision of services and facilities has a positive impact on the willingness of the population to engage in physical activity;

*H2*: User experience has a positive impact on the willingness of the population to engage in physical activity;

*H3*: The greenway landscape has a positive impact on the willingness of the population to engage in physical activity;

*H4*: Safety and accessibility have a positive impact on the willingness of the population to engage in physical activity;

*H5*: Personal and interpersonal factors have a positive impact on the willingness of the population to engage in physical activity.

### 3.3. Modifications to the structural equation model and results

**Figure 4:**
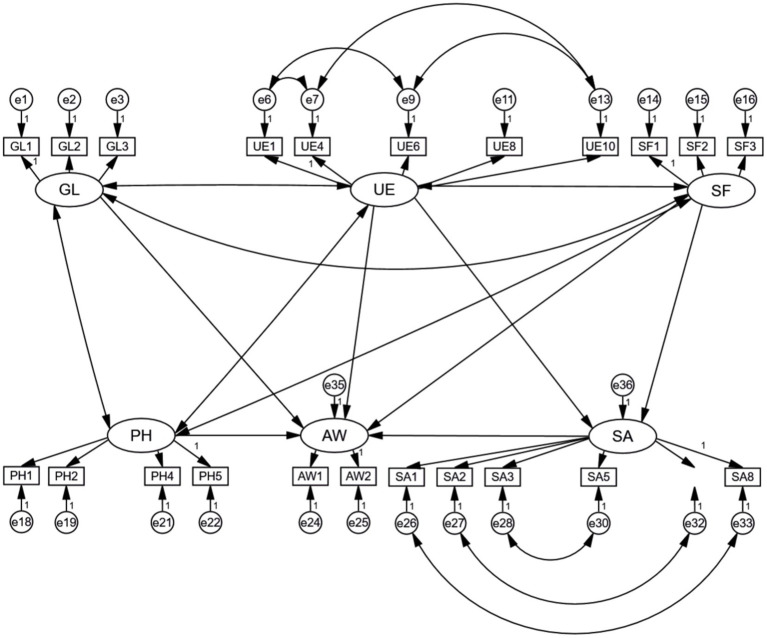
Structural equation model of factors restricting physical activity on Wenjiang Nancheng Greenway.

The modifications made to the structural equation model were mainly based on the MI (Modification Index) values and t values in the output results. In accordance with the principle of adjusting parameters in order of the associated MI value, from large to small, the observed variable corresponding to each of the relevant residual terms was removed or adjusted in turn under the premise of the model logic. In addition, under the premise that the model logic was reasonable, adjusted paths with a large MI value were added to analyze whether the adjustment was desirable by comparing the fit indices. After the above adjustments to and modifications of the initial model, each model fit index was significantly improved compared with the original model; the model fit is shown in Table 8. After these modifications, the chi-square value for the model was 968.364, with 210 degrees of freedom, and the *χ*^2^/DF ratio was 4.611, which is close to 3. Due to the large sample size of the questionnaire, the value was slightly higher, but still fell within acceptable limits. The RMSEA was close to 0.08, and the GFI, AGFI, CFI, IFI, and TLI were also close to 0.9. Again, due to the large sample size, the values deviated slightly, but they were all within the acceptable range. After modification, the overall fit of the model reached an acceptable standard, and a final structural equation model of the barriers to physical activity on the community greenway among the population was determined, as shown in Figure 4.

**Table 8:** Goodness of fit before and after model modification.

Goodness-of-fit measures	*χ*^2^/DF	GFI	CFI	RMSEA
Before model modification	6.699	0.633	0.673	0.133
Modified model	4.611	0.809	0.853	0.106

### 3.4. Structural equation model path analysis

According to the results of the analysis of the revised measurement model and structural model, the overall goodness of fit of the model was high, and the model was assumed to be reasonable in this study. Therefore, the strengths of the influence between variables could be evaluated using standardized path coefficients, and the research hypotheses proposed above could be tested and analyzed. The results indicated that H2, H3, and H4 were valid: that is, user experience (UE), the greenway landscape (GL), and safety and accessibility (SA) each had a positive impact on people’s willingness to engage in physical activity (AW), with path coefficients of 0.280, 0.205, and 0.163, respectively. However, H1 and H5 were not valid: that is, services and facilities (SF) and personal and interpersonal factors (PH) had no significant positive influence on willingness of the population to engage in physical activity (AW).

#### 3.4.1. Analysis of the weights of influence among variables in the structural model

By analyzing the path coefficients listed in Table 9, it can be seen that in the case of Wenjiang Greenway, Chengdu, China, the ranking of potential variables restricting people’s participation in physical activities in descending order of the weight of their influence was as follows: user experience (0.280) > the greenway landscape (0.205) > safety and accessibility (0.163). Therefore, on the whole, the “user experience” factor had the clearest restrictive effect on physical activity among the population, while the “safety and accessibility” factor had the least restrictive effect. The restrictive effects and influence of “services and facilities” and “personal and interpersonal factors” on physical activity among the population were inconsistent with the hypotheses. In addition, there was a positive correlation between the quality of “user experience” and “services and facilities” and the quality of “safety and accessibility,” with the influence weights of 0.168 and 0.630, respectively, indicating that the influence of user experience on safety and accessibility was greater.

**Table 9:** Path coefficients in the structural equation model.

Path between variables	Path coefficient
Safety and accessibility<−-- Services and facilities	0.168
Safety and accessibility<−--User experience	0.630
People’s willingness to engage in physical activity<−--Greenway landscape	0.205
People’s willingness to engage in physical activity<−--User experience	0.280
People’s willingness to engage in physical activity<−--Services and facilities	−0.263
People’s willingness to engage in physical activity<−--Personal and interpersonal factors	−0.190
People’s willingness to engage in physical activity<−--Safety and accessibility	0.163

#### 3.4.2. Analysis of the weights of influence among variables in the measurement model

**Figure 5:**
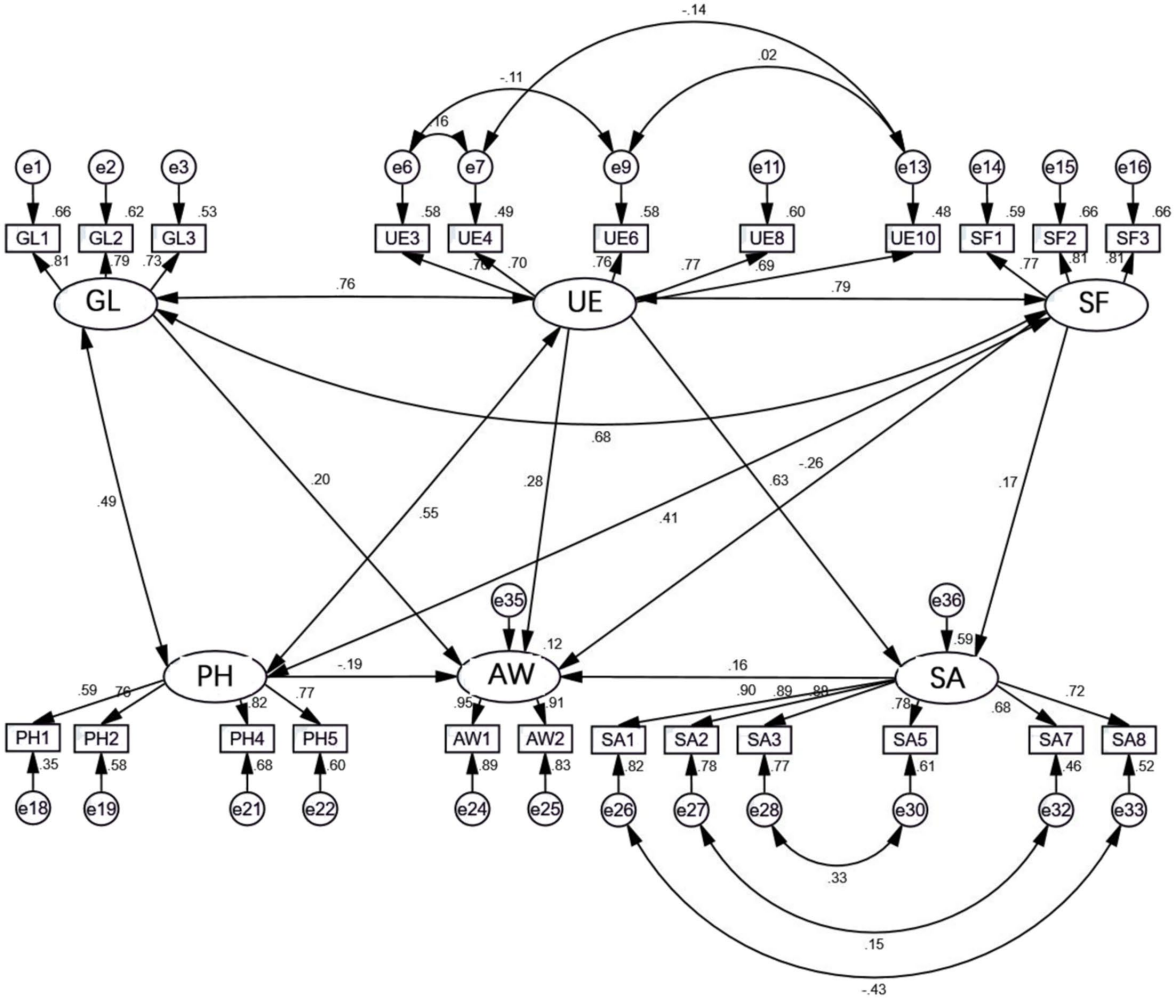
Path analysis in the model.

The weights representing the influence of the relationship between each observed variable and the corresponding latent variable can be seen in the model path analysis diagram (Figure 5), as shown in Table 10. Among items relating to the greenway landscape, the weight of influence of each item, ranked in descending order, was: architectural style and shape of gardens (0.810) > logo aesthetics (0.785) > planting of garden plants and shading effects (0.725). In terms of the user experience factor, the ranking of the weight of influence of each item was: ease of crossing of overpasses and tunnels (0.774) > marker lines indicate the correct condition (0.763) > barrier-free design (0.760) > signs and lines are simple and easy to understand (0.702) > pavement design (0.693). Within the factor of services and facilities, the ranking of the weight of influence of each item was: installation of streetlamps and other lighting (0.814) > installation and cleanliness of toilets and drinking fountains (0.812) > point-of-sale setup (0.771). Among the items relating to safety and accessibility, the ranking of the weight of influence of each item was: presence of sharp bulges in the seats and other facilities (0.904) > absence of unsafe remote dead ends (0.885) > installation of guardrails in hazard zones (0.878) > blocking the condition of motor vehicle facilities (0.783) > non-motor vehicle parking (0.724) > interference of pedestrians and bicycles with each other (0.677). Among the items falling under personal and interpersonal factors, the raking of the weight of influence of each item was: my physical health (0.822) > my recent physical condition (0.773) > attitudes of friends and family toward fitness (0.759) > my attitude toward fitness and sense of engagement (0.595). Finally, among items relating to people’s willingness to engage in physical activity, the weight of influence of overall satisfaction with the greenway (0.946) was greater than that of their willingness to go to the greenway for physical activity (0.911).

**Table 10:** Path coefficients in the measurement model.

Latent variable	Observed variable	Path coefficient
Greenway landscape (GL)	Architectural style and shape of gardens (GL1)	0.810
	Logo esthetics (GL2)	0.785
	Planting of garden plants and shading effect (GL3)	0.725
User experience (UE)	Marker lines indicate the correct condition (UE1)	0.763
	Signs and lines are simple and easy to understand (UE4)	0.702
	Barrier-free design (UE6)	0.760
	Ease of crossing of overpasses and tunnels (UE8)	0.774
	Pavement design (UE10)	0.693
Services and facilities (SF)	Point-of-sale setup (SF1)	0.771
	Installation and cleanliness of toilets and drinking fountains (SF3)	0.812
	Installation of streetlamps and other lighting (SF4)	0.814
Safety and accessibility (SA)	Presence of sharp bulges in the seats and other facilities (SA1)	0.904
	Absence of unsafe remote dead ends (SA2)	0.885
	Installation of guardrails in hazard zones (SA3)	0.878
	Blocking the condition of motor vehicle facilities (SA5)	0.783
	Interference of pedestrians and bicycles with each other (SA7)	0.677
	Non-motor vehicle parking (SA8)	0.724
Personal and interpersonal factors (PH)	My attitude toward fitness and sense of engagement (PH1)	0.595
	Attitudes of friends and family toward fitness (PH2)	0.759
	My physical health (PH4)	0.822
	Time occupied by work/family (PH5)	0.773
People’s willingness to engage in physical activity (AW)	Satisfaction with the greenway (AW1)	0.946
	Willingness to go to the greenway for physical activity (AW2)	0.911

## 4. Discussion

### 4.1. Objective constraints on the willingness of people to engage in physical activity on community greenways

In terms of objective environmental characteristics, previous studies have mostly explored the correlation between the environmental characteristics of public green spaces and their use for leisure activities from the perspective of promoting people’s engagement in leisure activities; in contrast, this study focused on the influencing factors and mechanisms that act as restrictions preventing people from using community greenways to engage in more physical activities. In this study, safety and accessibility were found to be important factors and mechanisms restricting people’s use of community greenways for physical activity. In terms of accessibility, the more distant and less accessible a community greenway is from where residents live, the lower the willingness of people to travel to the greenway for physical activity; reasonable organization of the flow of traffic and the availability of suitable parking spaces for private vehicles also affect residents’ willingness to travel. For instance, Lawrence et al. investigated the effect of urban greenway renovation on people’s engagement in physical activity and sedentary behavior, and concluded that accessibility is an important factor in enhancing people’s willingness to engage in physical activity (51); some scholars have also shown that as distance increases, the frequency of green space use decreases, leading to a decrease in the probability of residents’ using green space for physical activity and their willingness to do so (52–54). In terms of safety, existing studies have focused on the differences between different types of environments in terms of the perceived safety of users (55) and the correlation between the configuration of visual elements and users’ perceived safety (56), while some scholars have confirmed through empirical studies that safety can have a significant impact on people’s willingness to engage in physical activity and on the benefits of physical activity (57, 58). In this study, it was found that the presence of hidden safety risks at recreational sports facilities, a low sense of security created by activity spaces, and imperfect safety measures in dangerous areas are important barriers to use of community greenways for physical activities. This study also revealed the aspects of the greenway landscape that act as restrictions to people’s use of community greenways for physical activities and the mechanisms by which these restrictions act. A single type of greenway landscape and poor quality of the landscape environment are important factors that restrict people’s access to greenways for physical activity. Zhang and colleagues explored the influence of the greenway landscape environment on users’ leisure activities through machine vision, but their study found that the environmental characteristics of the greenway do not affect the distribution of leisure activities engaged in (59). In addition, this study found that when the greenway landscape does not reflect the esthetic philosophy and values of the users, this will greatly constrain users’ willingness to engage in physical activity. The findings of this study are similar to those of previous studies: for example, Junga et al. demonstrated that greenway landscape features are positively associated with users’ perceived and preferred experiences, thereby influencing users’ willingness to engage in physical activity (60). Similarly, Bao et al. showed that satisfying the demand for physical activity in neighborhood spaces through the proper configuration of neighborhood spatial and environmental features is conducive to enhancing people’s willingness to engage in physical activity (61). According to the results of this study, services and facilities do not have a constraining effect on people’s willingness to engage in physical activity in community greenways: i.e., the installation and distribution of services and facilities in community greenways does not restrict people’s willingness to engage in physical activity in these spaces. This contradicts previous studies, which have shown that both greening rates and the number of neighborhood fitness facilities can promote residents’ engagement in recreational physical activity and willingness to engage in physical activity (62). For example, Zhai et al. explored the effect of the configuration of park facilities on the intensity of physical activity among older adults, and the study confirmed a significant correlation between the intensity of physical activity and the type and quality of the configuration of park facilities (63). Additionally, Jenny et al. demonstrated that the upgrading of park services helps to increase visitors’ willingness to engage in physical activity and the vitality of the spatial environment (62).

### 4.2. Subjective constraints on the willingness of people to engage in physical activity on community greenways

In terms of subjective personal factors, previous studies have mostly started from the personal characteristics of users to explore the relationship between their personal attributes, such as gender, age, and occupation (64), and the use of green public spaces; in contrast, this study explored the mechanisms underlying factors hindering users’ willingness to engage in physical activity from the perspective of users’ physiological, psychological, and interpersonal characteristics. This study found that there was no inhibitory relationship between the personal and interpersonal characteristics of users and people’s intentions to engage in physical activity on community greenways, indicating that users’ individual characteristics and interpersonal relationships did not act as restrictions preventing them from going to the greenway for physical activity. This is not in line with the results of existing studies: in terms of the individual characteristics of residents, it has been demonstrated that older adults are more likely to exercise on greenways than younger people, and that greenways have important health benefits for middle-aged and older adults who are exposed to health risks. In addition, residents with higher levels of education, higher annual income, and good health status use greenways more frequently and report higher willingness to engage in physical activity, so these characteristics are likely to promote the improvement of physical activity levels through the use of greenways (65). In terms of peer relationships, Zhu et al., in considering the moderating effects of social support in the relationship between neighborhood green spaces and residents’ engagement in physical activity as a form of leisure, found that an increase in the number of exercise-loving friends among residents would only enhance the positive effect of greening rate, but in turn would weaken the positive effect of increasing the availability of fitness facilities on the degree of engagement in physical activity for leisure (66). This study also revealed that personal experience of greenway use is an important factor that hinders users’ willingness to engage in physical activity. Improving user experience and satisfaction with public space can enhance users’ willingness to engage in physical activity (67). Zhao et al. demonstrated that the subjective perception of humanized space has a direct impact on the duration of physical activity among the public, while the connectivity of the destination and the degree to which the landscape is maintained have an indirect impact on the level of physical activity among the public via the subjective perceptions and user experience of users (68).

## 5. Conclusion

With a focus on restrictions, this study explored the factors influencing people’s willingness to engage in physical activity on community greenways, indicating that community greenways can provide support for community residents in the form of a space for them to carry out healthy physical activity and daily leisure activities; however, there are also many factors restricting such participation. Adopting a field investigation methodology along with theoretical modeling of hypotheses regarding the factors restricting people’s use of community greenways for physical activity, an empirical study was conducted, taking Chengdu community greenways as the object of research to explore the correlations of the landscape, the safety and accessibility of greenways, and the user experience with people’s intentions to engage in physical activity. The results of the analysis of the effects of services and facilities, and of individual and interpersonal factors, showed that although the data observed were inconsistent with the hypotheses, these factors still have certain constraining effects on people’s willingness to engage in physical activity.

This study has several limitations. First, the empirical investigation reported in this article was conducted in the early winter season. Due to the specific limitations of the season, the number and type of interviewees was insufficient, and the collected data (and thus the results of the analysis) were not sufficiently representative enough of users during other seasons. In addition, all questionnaires were designed with the support of a large body of literature, including expert interviews and pre-research. However, the ways in which certain items were expressed in the final questionnaire may have been slightly obscure to some non-professionals, resulting in incomplete understanding of the full intention of some items of the questionnaire.

## Data availability statement

The original contributions presented in the study are included in the article/Supplementary material, further inquiries can be directed to the corresponding authors.

## Author contributions

EF provided the research ideas and formulated the overall research objectives, reviewed the literature in the early stage of the experiment, participated in the research and investigation process during the research process, guided and participated in the article writing process. XD conducted the field survey, participated in the analysis of the questionnaire and the data, and was responsible for writing the results, discussion and summary of the paper. YW prepared the preliminary questionnaire, and participated in the field survey and the distribution, sorting and analysis of the questionnaire. LL participated in the field investigation of this study and the writing and translation of the article. YX participated in the field investigation and writing and translation of the study. ML participated in the writing and translation of the article. ZZ participated in the writing and translation of the article. JD guided the writing of this paper and provided help with the research methods and ideas. XnL participated in the writing and translation of the study, and participated in the experimental preparation process of the study. XiL provided theoretical guidance for this study and provided funding.

## Conflict of interest

The authors declare that the research was conducted in the absence of any commercial or financial relationships that could be construed as a potential conflict of interest.

## Publisher’s note

All claims expressed in this article are solely those of the authors and do not necessarily represent those of their affiliated organizations, or those of the publisher, the editors and the reviewers. Any product that may be evaluated in this article, or claim that may be made by its manufacturer, is not guaranteed or endorsed by the publisher.
